# Combined inhibition of Bcl-2/Bcl-xL and Usp9X/Bag3 overcomes apoptotic resistance in glioblastoma *in vitro* and *in vivo*

**DOI:** 10.18632/oncotarget.3993

**Published:** 2015-05-04

**Authors:** Georg Karpel-Massler, Chang Shu, Lily Chau, Matei Banu, Marc-Eric Halatsch, Mike-Andrew Westhoff, Yulian Ramirez, Alonzo H. Ross, Jeffrey N. Bruce, Peter Canoll, Markus D. Siegelin

**Affiliations:** ^1^ Department of Pathology and Cell Biology, Columbia University Medical Center, New York, USA; ^2^ Department of Neurosurgery, Columbia University Medical Center, New York, USA; ^3^ Department of Neurosurgery, Ulm University Medical Center, Ulm, Germany; ^4^ Department of Pediatrics and Adolescent Medicine, Ulm University Medical Center, Ulm, Germany; ^5^ Department of Biochemistry and Molecular Pharmacology, University of Massachusetts Medical School, Massachusetts, USA

**Keywords:** glioblastoma, BH3-mimetic, apoptotic resistance, ABT263, GX15-070

## Abstract

Despite great efforts taken to advance therapeutic measures for patients with glioblastoma, the clinical prognosis remains grim. The antiapoptotic Bcl-2 family protein Mcl-1 is overexpressed in glioblastoma and represents an important resistance factor to the BH-3 mimetic ABT263.

In this study, we show that combined treatment with ABT263 and GX15-070 overcomes apoptotic resistance in established glioblastoma cell lines, glioma stem-like cells and primary cultures. Moreover, this treatment regimen also proves to be advantageous *in vivo*. On the molecular level, GX15-070 enhanced apoptosis by posttranslational down-regulation of the deubiquitinase, Usp9X, and the chaperone Bag3, leading to a sustained depletion of Mcl-1 protein levels. Moreover, knock-down of Usp9X or Bag3 depleted endogenous Mcl-1 protein levels and in turn enhanced apoptosis induced through Bcl-2/Bcl-xL inhibition.

In conclusion, combined treatment with ABT263 and GX15-070 results in a significantly enhanced anti-cancer activity *in vitro* as well as *in vivo* in the setting of glioblastoma. Both drugs, ABT263 and GX15-070 have been evaluated in clinical studies which facilitates the translational aspect of taking this combinatorial approach to the clinical setting. Furthermore we present a novel mechanism by which GX15-070 counteracts Mcl-1 expression which may lay a foundation for a novel target in cancer therapy.

## INTRODUCTION

The prognosis for patients with glioblastoma is grim [1]. Great efforts have been taken to improve therapeutic means and thus survival. However, since the introduction of temozolomide, no further therapeutic approach followed to enter clinical application and to lead to a substantial improvement of the fate of glioma patients. This fact also holds true for many targeted therapies that were based on the identification of oncogenes, which were found to be frequently dysregulated in this disease [2-5]. The highly heterogeneous nature of glioblastoma and mechanisms of inherent or acquired resistance such as cross talk between different signaling pathways are likely to largely contribute to this phenomenon [6]. Therefore, strategies overcoming mechanisms of resistance are a logical and urgently needed step to advance the development of more efficient therapeutic approaches.

The B-cell lymphoma-2 (Bcl-2) family proteins are important regulators of apoptosis and consist of pro- and anti-apoptotic members [7]. Dysregulation of this balanced system represents an important feature in cancer [8]. As a consequence targeted agents were developed to manipulate an anti-apoptotic dysbalance in the sense of shifting the cellular homeostasis of cancer cells towards apoptosis. ABT-compounds such as ABT737 or ABT263 target the anti-apoptotic Bcl-2 family proteins Bcl-2, Bcl-xL and Bcl-w and promote apoptosis by inhibiting the sequestration of the pro-apoptotic multidomain effectors BAX and BAK which subsequently oligomerize leading to mitochondrial outer membrane permeabilization and the release of cytochrome c into the cytosol.

However, presence or even compensatory up-regulation of myeloid cell leukemia 1 (Mcl-1), another anti-apoptotic member of the Bcl-2 family, was shown to confer resistance towards the treatment with ABT-compounds [9, 10]. In addition, *in silico* analysis based on the TCGA data set revealed a 5-fold overexpression of Mcl-1 mRNA in glioblastoma when compared to normal brain (Supplementary Figure 1) suggesting that Mcl-1 represents a valid target in this disease. GX15-070 is a small-molecule inhibitor of Mcl-1 and was shown to overcome Mcl-1-mediated resistance in oral carcinoma and murine melanoma cells [11]. Both, ABT-compounds as well as GX15-070, mimic the role of pro-apoptotic Bcl-2 family proteins such as BAD, NOXA or PUMA sharing a conserved dimerization motif called Bcl-2 homology 3 (BH3).

With this study, we examined whether combined treatment with the two BH3-mimetics ABT263 and GX15-070 may represent a valuable therapeutic approach in the setting of glioblastoma. Our data show that the combination therapy with ABT263 and GX15-070 yields a synergistic antiproliferative effect on established and glioma stem-like cells. In addition, in a heterotopic murine xenograft model this combination treatment resulted in a significant reduction in tumor size when compared to single-agent treatments or control and therefore holds promise as a novel therapeutic strategy in this disease. On the molecular level, we show that down-regulation of the Mcl-1 chaperone Bcl-2-associated athanogene domain 3 (Bag3) and the deubiquitinase Ubiquitin-specific peptidase 9, X-linked (Usp9X) represent a novel mechanism responsible for GX15-070-mediated down-regulation of Mcl-1.

## RESULTS

### Combined treatment with ABT263 and GX15-070 yields a synergistic antiproliferative effect

**Figure 1:**
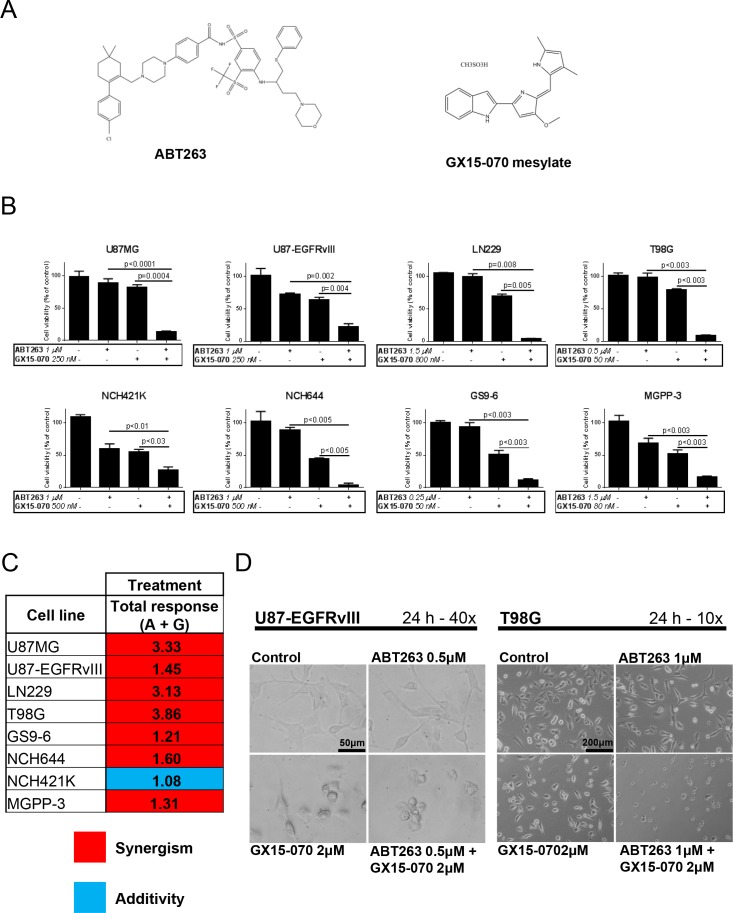
Combined treatment with ABT263 and GX15-070 results in a synergistic antiproliferative effect across the majority of establishe glioma stem-like and primary cultured glioblastoma cells **A.** chemical structures of ABT263 and GX15-070. **B.** U87MG, U87-EGFRvIII, LN229, T98G, glioma stem-like cells NCH421K, NCH644 and two glioblastoma primary cultures (human GS9-6 and murine MGPP-3) were treated with the indicated concentrations of ABT263 and GX15-070 or both agents under serum starvation (1.5% FBS). After 72 h, a MTT assay was performed. Data presented are representative for at least two independent experiments. Error bars: standard error of the mean (SEM). **C.** consolidated representation of qualitative combined antiproliferative effects of ABT263 (A) and GX15-070 (G) on established glioma stem-like and primary cultured glioblastoma cells. The antiproliferative effect of the different drug combinations was assessed by an MTT assay prior to performing Bliss analysis in order to determine whether the combined treatments yielded synergisti, **c:** additive or antagonistic effects. The expected total response to the combination treatment was calculated as fractional response to drug A (*F*_a_) + fractional response to drug B (*F*_b_) - *F*_a_ x *F*_b_. If the ratio of the actual total response and the expected total response equaled 0.9 to 1.1, additivity was assumed. If this quotient was less than 0.9, the effect was described as antagonistic. Synergism was stated if the quotient was greater than 1.1. **D.** representative microphotographs of U87-EGFRvIII and T98G cells at indicated magnifications after 24 h of treatment with ABT263, GX15-070 or both. Morphological changes such as a rounding of cells are commonly seen after treatment with both agents.

To test our hypothesis that combined treatment with the Bcl-2 inhibitor ABT263 (Figure 1A) and the Mcl-1 inhibitor GX15-070 (Figure 1A) yields a more favorable antineoplastic activity in glioblastoma when compared to single-agent treatments, we first examined effects on cellular proliferation by MTT assays. As shown in Figure 1B, combined treatment with ABT263 and GX15-070 resulted in a marked cooperative antiproliferative effect for all tested glioblastoma cells. Statistical analysis using the Bliss equation revealed that, except for NCH421K, in all other established glioblastoma cell lines, glioma stem-like cells or primary cultures, the combination therapy with ABT263 and GX15-070 inhibited proliferation in a synergistic manner (Figure 1C). In NCH421K, the combination therapy yielded an additive effect.

### GX15-070 enhances the pro-apoptotic effect of ABT263 on cellular and molecular levels

**Figure 2:**
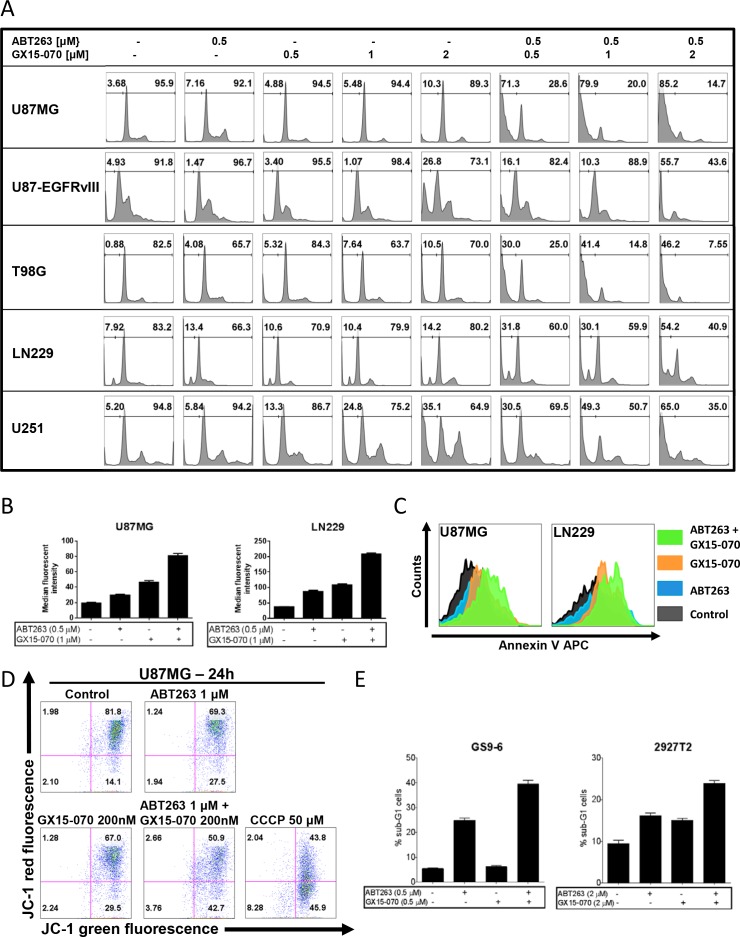
Combined treatment with ABT263 and GX15-070 results in an enhanced induction of apoptosis **A.** representative histograms of U87MG, U87-EGFRvIII, T98G, LN229 and U251 glioblastoma cells that were treated for 48 h with the indicated concentrations of ABT263, GX15-070, both, or solvent prior to staining with propidium iodide and flow cytometric analysis. **B.** quantitative representation of annexin V-positive U87MG and LN229 glioblastoma cells treated for 72 h with ABT263, GX15-070, both, or solvent at indicated concentrations. Columns, means of three serial measurements. Bars, SD. **C.** representative histograms of annexin V-positive U87MG and LN229 glioblastoma cells treated as described for Figure 2B. **D.** representative histograms of U87MG cells that were treated for 24 h with ABT263, GX15-070, both, or solvent prior to staining with JC-1 and flow cytometric analysis. Cells treated with the mitochondria depolarizing agent carbonylcyanide *m*-chlorophenylhydrazone (CCCP) served as a positive control. **E.** quantitative representation of the fraction of sub-G1 cells. GS9-6 and 2927T2 glioblastoma primary cultures were treated and analyzed as described for Figure 2A. Columns, means of three serial measurements. Bars, SD.

As microscopic imaging showed, the antiproliferative effect of the combination treatment was accompanied by another prominent finding. Cellular morphology was observed to be markedly changed in cells treated with both agents displaying a reduction of cytoplasmic processes as the most pronounced feature (Figure 1D). The morphological appearance pointed towards apoptosis as mechanism. To clarify this suspicion, we performed staining for propidium iodide and flow cytometric analysis. Combined treatment with ABT263 and GX15-070 resulted in a dose-dependent increase of the sub-G1 fraction (apoptotic cells) of U87MG, U87-EGFRvIII, T98G, LN229 and U251 glioblastoma cells when compared to single-agent treatment or control (Figure 2A and Supplementary Figure 2). Similar results were found in two glioblastoma primary cultures - GS9-6 and 2927T2 (Figure 2E). Staining for annexin V APC was performed, further confirming the enhancement of apoptosis by the combination therapy using an independent and more apoptosis-specific method (Figure 2B and 2C). To address the question whether the intrinsic pathway of apoptosis is involved, changes to the mitochondrial membrane potential were examined. Our data show that the combined treatment yields an enhanced reduction of the mitochondrial membrane potential in U87MG cells when compared to cells treated with either agent alone or solvent thus, underlining the role of intrinsic apoptosis (Figure 2D).

**Figure 3:**
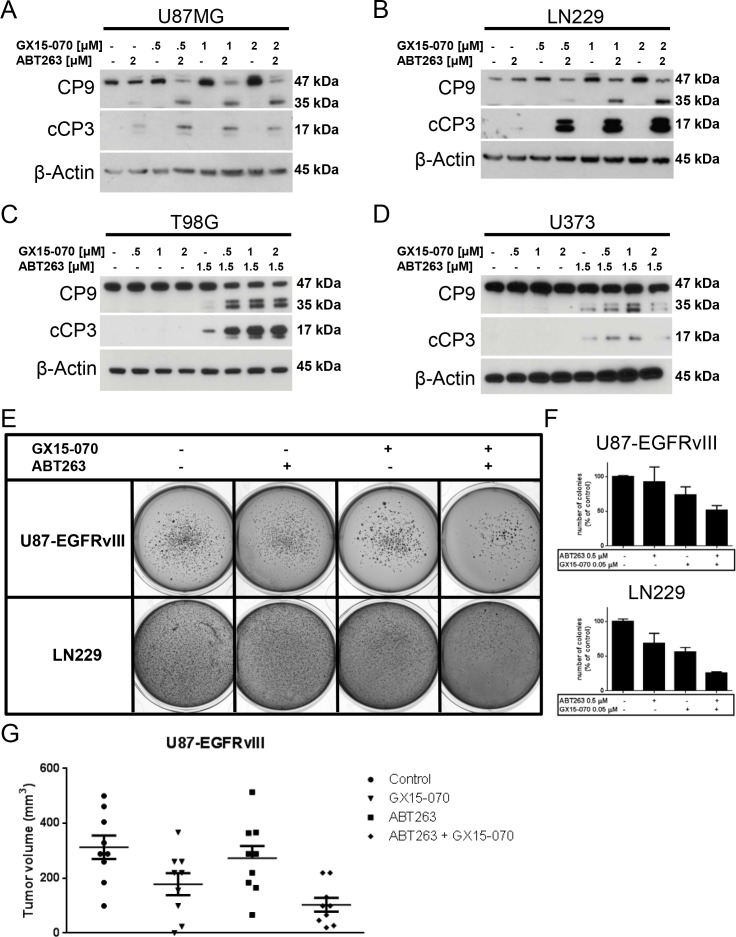
Combined treatment with ABT263 and GX15-070 results in enhanced cleavage of caspases 9 and 3 and enhanced inhibition of anchorage-independent growth as well as *in vivo* tumor formation **A.**-**D.** U87MG **A.** LN229 **B.** T98G **C.** and U373 **D.** glioblastoma cells were treated for 7 h with ABT263, GX15-070, both agents or solvent under serum starvation. Whole-cell extracts were examined by Western blot for caspase 9 (C9) and cleaved caspase 3 (cC3). Actin Western blot analysis was performed to confirm equal protein loading. **E.** U87-EGFRvIII and LN229 cells were grown in 0.35% agarose in DMEM supplemented with 10% FBS for 21 d. Representative photographic images are shown. **F.** quantitative representation of the number of colonies formed. Only colonies with an area > 625 μm^2^ were counted. Experiments were done in duplicate and repeated once (*n* **=** 2). Columns, mean of the number of colonies counted for one representative experiment. Bars, SEM. **G.** U87-EGFRvIII cells were pretreated as indicated in the materials and methods section and implanted subcutaneously in mice. Scatter plot showing 9 tumors per group. Line, mean; bars, SEM.

After having shown that the combination therapy with ABT263 and GX15-070 markedly enhances the induction of apoptosis on the cellular level we examined the response at the molecular level. Therefore, Western blot analyses for activation of caspases 9 and 3 were conducted in U87MG, LN229, T98G and U373 glioblastoma cells. In all these cell lines, the combination therapy resulted in markedly enhanced cleavage of initiator caspase 9 and effector caspase 3 (Figure 3A-3D).

### GX15-070 enhances the inhibitory effect of ABT263 on colony-forming ability *in vitro* and on tumor formation *in vivo*

Next, we investigated whether the combination therapy is superior in terms of inhibiting anchorage-independent growth. For this purpose, a soft agar assay was used in which U87-EGFRvIII and LN229 cells were treated with 1 μM ABT263, 0.5 μM GX15-070, both agents or solvent. After 21 days of continuous drug exposure, representative images were taken and analysed. Only colonies larger than 625 μm^2^ were counted. Typically, both cell lines formed a high number of colonies under control conditions (Figure 3E and 3F). While treatment with either ABT263 or GX15-070 alone resulted in minor reductions of colony formation, concomitant treatment with both compounds greatly reduced anchorage-independent growth in both cell lines (Figure 3E and 3F). This effect was less pronounced in U87-EGFRvIII cells that harbor the constitutively active EGFRvIII and have a particularly resistant phenotype.

Further, we examined whether combined treatment with GX15-070 and ABT263 also impairs tumor formation in an *in vivo* setting that allows for an interaction between the tumor and surrounding tissue, including neoplastic features such as neo-angiogenesis. Therefore, we implanted pretreated U87-EGFRvIII cells subcutaneously into the flanks of SCID SHO mice and determined tumor volumes once tumors formed. As outlined in Figure 3G, combined treatment with ABT263 and GX15-070 did not prevent tumor formation but resulted in a marked reduction of tumor volumes.

### Treatment with GX15-070 results in down-regulation of the Mcl-1-chaperone Bag3 and subsequent loss of Mcl-1 and Bcl-2

**Figure 4:**
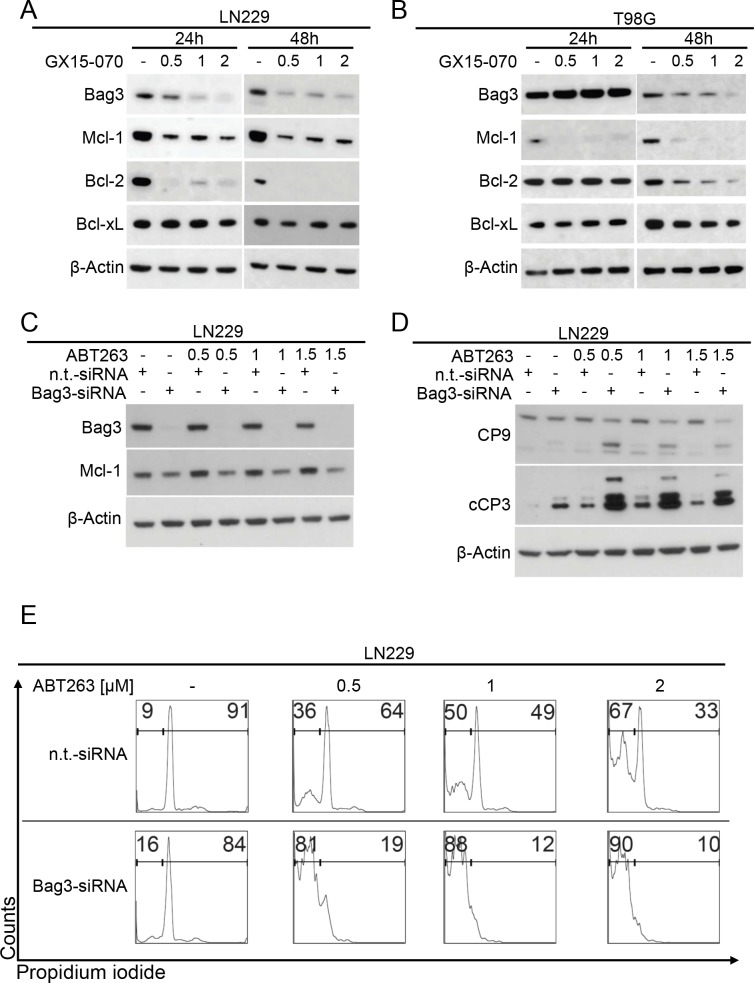
Down-regulation of Bag3 sensitizes for ABT263-mediated apoptosis **A.**-**B.** LN229 **A.** and T98G **B.** glioblastoma cells were treated for 24 and 48 h with increasing concentrations of GX15-070. Whole-cell extracts were examined by Western blot for Bag3, Mcl-1, Bcl-2 and Bcl-xL. Actin Western blot analysis was performed to confirm equal protein loading. **C.** LN229 glioblastoma cells were treated with Bag3-siRNA or non-targeting (n.t.)–siRNA as described in the materials and methods section prior to treatment with ABT263 at indicated concentrations for 7 h. Whole-cell extracts were examined by Western blot for Bag3 and Mcl-1. Actin served as loading control. **D.** LN229 glioblastoma cells were treated as described for Figure 4C prior to collecting whole-cell extracts and performing Western blot analysis for caspases 9 (CP9) and 3 (cCP3). **E.** representative histograms of LN229 glioblastoma cells treated with n.t.-siRNA or Bag3-siRNA prior to treatment with ABT263 for 24 h. Then cells were stained for PI and subjected to flow cytometric analysis. The fraction of sub-G1 cells was determined.

Next, we dissected the molecular events responsible for the GX15-070-mediated sensitization towards ABT263. It is known that GX15-070 inhibits Mcl-1 [11]. However, the exact mechanism is not fully understood. Bag3 has been shown to stabilize Mcl-1 [12]. In the same study, silencing of Bag3 increased the response to ABT263 in different cancer cell lines not including gliomas. Our data show that treatment with GX15-070 results in a dose-dependent down-regulation of Bag3 in LN229 and T98G glioblastoma cells (Figure 4A and 4B). Moreover, this finding is accompanied by a pronounced down-regulation of Mcl-1 and Bcl-2, suggesting that GX15-070-mediated down-regulation of Bag3 leads to loss of Mcl-1 and Bcl-2 (Figure 4A and 4B). Notably, the expression of Bcl-xL was not altered.

### Down-regulation of Bag3 sensitizes for ABT263-mediated apoptosis by decreasing Mcl-1 protein levels in glioblastoma cells

To address the question whether down-regulation of Bag3 by GX15-070 sensitizes glioblastoma cells towards ABT263-mediated apoptosis, we performed knock-down experiments silencing Bag3 prior to treatment with increasing concentrations of ABT263. Treatment with Bag3-siRNA resulted in an efficient down-regulation of Bag3 expression in LN229 cells (Figure 4C). In addition, up-regulation of the anti-apoptotic Bcl-2 family protein Mcl-1, following treatment with ABT263, was counteracted by silencing Bag3. Moreover, knock-down of Bag3 markedly enhanced cleavage of caspases 9 and 3 upon treatment with ABT263, compared to cells subjected to single- or combined treatment with non-targeting siRNA and ABT263 (Figure 4D). These findings were confirmed on the cellular level by staining with PI and subsequent flow cytometric analysis. Silencing of Bag3 combined with ABT263-treatment significantly increased the fraction of sub-G1 cells compared to cells treated with non-targeting siRNA plus ABT263 or Bag3-siRNA alone (Figure 4E).

To examine whether down-regulation of Mcl-1 is sufficient to sensitize glioma cells for ABT263-mediated apoptosis, we performed knock-down experiments targeting Mcl-1 in LN229 glioblastoma cells. Silencing Mcl-1 alone resulted in a marked decrease but not a total suppression of Bag3 (Supplementary Figure 3A). However, knock-down of Mcl-1 combined with treatment with ABT263 yielded a complete suppression of Bag3. This finding was accompanied by an enhanced cleavage of caspases 9 and 3 as well as PARP and an increase in the fraction of sub-G1 cells (Supplementary Figure 3B and C). Silencing of Mcl-1 alone resulted in a greater increase of the fraction of sub-G1 cells compared to silencing of Bag3 alone, suggesting that down-regulation of Mcl-1 is ultimately the critical event for enhancement of responses to ABT263, including apoptosis. Again, expression of Bcl-xL was not markedly altered.

### Treatment with GX15-070 results in down-regulation of the deubiquitinase Usp9X

**Figure 5:**
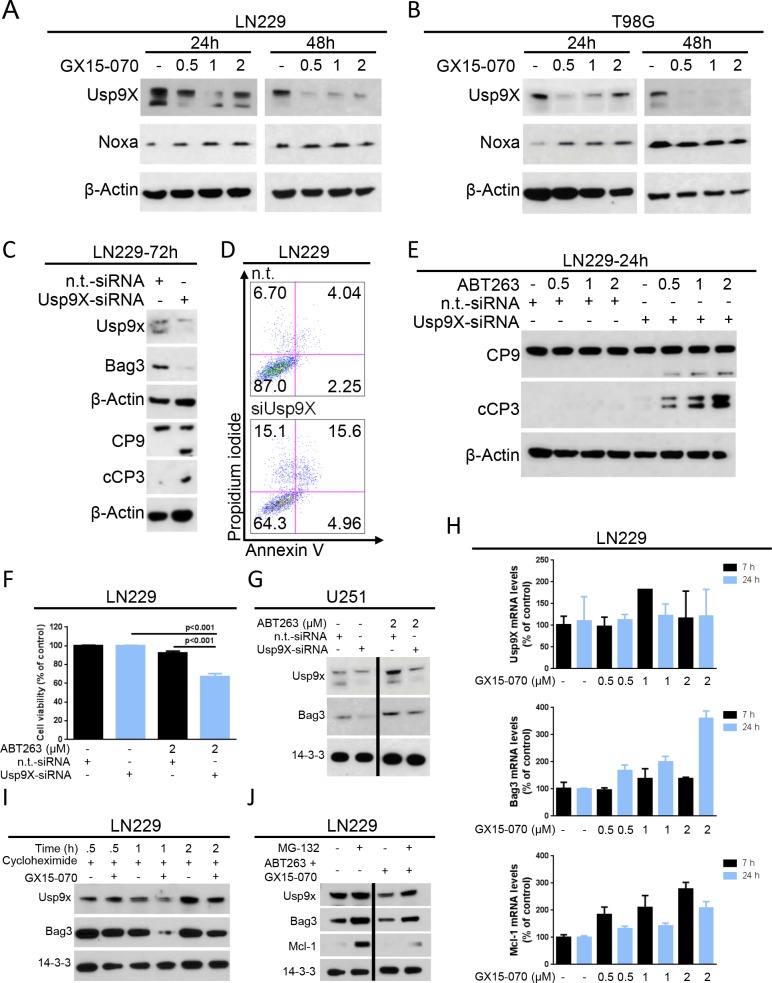
GX15-070 down-regulates the deubiquitinase Usp9X which sensitizes cells for ABT263-mediated apoptosis **A.**-**B.** LN229 **A.** and T98G **B.** glioblastoma cells were treated for 24 and 48 h with increasing concentrations of GX15-070. Whole-cell extracts were examined by Western blot for Usp9X and Noxa. Actin Western blot analysis was performed to confirm equal protein loading. **C.** LN229 glioblastoma cells were treated with Usp9X-siRNA or non-targeting (n.t.)–siRNA prior to Western blot analysis for Usp9X, Bag3, caspase 9 (C9) and cleaved caspase 3 (cC3). Actin Western blot analysis was performed to confirm equal protein loading. **D.** representative histograms of LN229 glioblastoma cells treated with Usp9X-siRNA or n.t.–siRNA prior to staining with annexin V/PI and flow cytometric analysis. **E.** LN229 glioblastoma cells were treated with Usp9X-siRNA or n.t.–siRNA prior to treatment with ABT263 at indicated concentrations for 24 h. Whole-cell extracts were examined by Western blot for caspase 9 (C9) and cleaved caspase 3 (cC3). Actin served as a loading control. **F.** LN229 glioblastoma cells were transfected either with Usp9X-siRNA or n.t.–siRNA. Treatment with ABT263 or solvent was performed for 24 h prior to staining for annexin V/PI and flow cytometric analysis. Columns, means of viable cells. Bars, SEM. **G.** U251 glioblastoma cells were treated either with Usp9X-siRNA or n.t.–siRNA. Treatment with ABT263 or solvent was performed for 48 h. Whole-cell extracts were examined by Western blot for Usp9X and Bag3. 14-3-3 Western blot analysis was performed to confirm equal protein loading. **H.** LN229 cells were treated for 7 or 24 h with increasing concentrations of GX15-070 or solvent. Cells were collected and RNA isolated prior to performing rtPCR for Usp9X, Bag3 and Mcl-1. **I.** LN229 glioblastoma cells were treated with the protein synthesis inhibitor cycloheximide (10 μg/ml) in the presence or absence of GX15-070 (1 μM) for the indicated time points. Western blot analysis was performed for Usp9X and Bag3. 14-3-3 expression was determined to confirm equal protein loading. **J.** LN229 glioblastoma cells were treated with or without the proteasome inhibitor MG132 (10 μM) in the presence or absence of the combination of ABT263 (0.5 μM) and GX15-070 (1 μM) for 5 h. Western blot analysis was performed for Usp9X, Bag3 and Mcl-1. 14-3-3 expression was determined to confirm equal protein loading.

Usp9X modulates Mcl-1 levels and counteracts apoptosis in cancer cells [13]. Given that GX15-070 suppresses Mcl-1, we examined whether it regulates the expression of Usp9X. In LN229 and T98G cells, we detected a strong reduction in Usp9X levels after 24 h and 48 h of treatment with increasing concentrations of GX15-070 (Figure 5A-5B). In contrast, Noxa levels revealed a slight up-regulation in T98G cells treated with GX15-070 (Figure 5B). Next we silenced Usp9X in LN229 glioblastoma cells to determine the impact of Usp9X on the chaperone Bag3 as well as initiator- and effector caspases. Silencing of Usp9X by siRNA results in down-regulation of Usp9X protein levels, concomitant suppression of Bag3, strong activation of caspase-9/-3 and apoptosis induction (Figure 5C-5D), suggesting that suppression of Usp9X is a pivotal regulator of intrinsic apoptosis and a potent sensitizer for intrinsic apoptotic stimuli in *TP53*-mutated glioblastoma cells. Consistently, LN229 cells with silenced Usp9X showed a greater sensitivity to ABT263 as evidenced by enhanced activation of caspase-9/-3 and a pronounced reduction in cellular viability compared to LN229 cells transfected with non-targeting siRNA (Figure 5E-5F). Since suppression of Usp9X significantly enhances apoptosis induced by ABT263, we determined the effects of ABT263 on Usp9X and Bag3 protein levels. U251 cells were transfected with non-targeting or Usp9X-specific siRNA and treated with 2 μM ABT263 (Figure 5G). While U251 cells transfected with non-targeting siRNA showed an increase in both Usp9X and Bag3 protein levels after treatment with ABT263, this induction was attenuated in cells transfected with Usp9X-siRNA (Figure 5G). Thus, ABT263-mediated induction of both Usp9X and Bag3 is inhibited by interference with Usp9X levels, suggesting that Usp9X is upstream of Bag3 and that it is implicated in the regulation of its expression. Next, we examined how GX15-070 regulates the expression levels of both Usp9X and Bag3. Therefore, LN229 cells were treated with cycloheximide in the presence or absence of GX15-070. LN229 cells treated with the combination of cycloheximide and GX15-070 showed a significantly lower expression of both Bag3 and Usp9X, indicating that GX15-070 most likely regulates Bag3 and Usp9X in a posttranslational manner, potentially involving enhanced proteasomal degradation (Figure 5I). To explore the mechanisms of enhanced proteasomal degradation LN229 cells were treated with MG132, ABT263/GX15-070 or the triple therapy of ABT263/GX15-070/MG132. While the combination of ABT263 and GX15-070 depleted both Usp9X and Bag3 levels, the triple therapy partially rescued the expression of Usp9X and Bag3 (Figure 5J). To determine whether GX15-070 regulates Usp9X, Bag3 or Mcl-1 expression on the transcriptional level, we conducted real-time PCR analyses and found that in LN229 cells Usp9X mRNA levels were only minimally affected by GX15-070 at the indicated time points (Figure 5H). Bag3 and Mcl-1 mRNA levels were markedly increased by GX15-070 treatment in LN229 cells indicating a compensatory response. Overall, down-regulation of Usp9X, Bag3 and Mcl-1 on the protein level does not seem to be transcriptionally mediated.

### Treatment with GX15-070 enhances the antineoplastic effect of ABT263 *in vivo*

**Figure 6:**
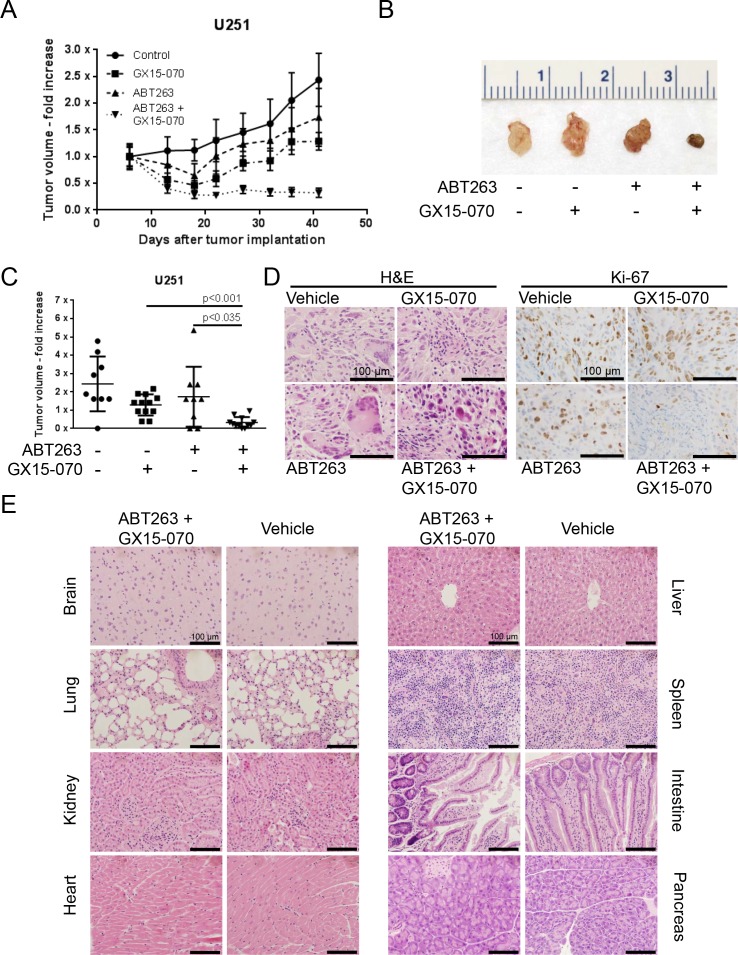
Combined treatment with ABT263 and GX15-070 results in an enhanced inhibition of tumor growth *in vivo* 1×10^6^ U251 glioblastoma cells were implanted subcutaneously. After tumor formation animals were treated intraperitoneally with vehicle (*n* = 9 tumors), GX15-070 (3 mg/kg; *n* = 12 tumors), ABT263 (50 mg/kg; *n* = 9 tumors) or both agents (*n* = 12 tumors) twice a week for 3 weeks. Data are presented as mean and SEM. **A.** tumor growth curves showing the fold increase in tumor size for each treatment group. **B.** representative photographs of the tumors. **C.** quantification and statistical analysis of the different treatment groups 41 days after tumor implantation. The Mann-Whitney *U*-test was used for statistical analysis and a *p*-value of less than 0.05 was considered statistically significant. **D.** microphotographs showing the histological morphology of representative tumors treated as indicated after staining for hematoxylin and eosin (H&E left panel) or Ki-67 (right panel). Magnification, x40; scale bar, 100 μm. **E.** representative microphotographs showing the histological morphology (H&E staining) of the indicated organs among representative animals receiving treatment either with vehicle or the combination of ABT263 and GX15-070. Magnification, x40; scale bar, 100 μm.

To assess the therapeutic efficacy of this combination therapy *in vivo*, U251 glioblastoma cells were implanted subcutaneously. Once tumors formed, the mice were randomized and treated with ABT263 (50 mg/kg), GX15-070 (3 mg/kg), both agents or solvent. Under these conditions, animals that received the combination treatment had statistically significantly smaller tumors than animals treated with vehicle, ABT263 or GX15-070 alone (Figure 6A-6C). Remarkably, animals treated with the drug combination showed tumor regression, indicating that the combination therapy did not only affect tumor proliferation it also impacted tumor cell death *in vivo* (Figure 6A). On histological sections we found that tumors treated with ABT263/GX15-070 displayed a higher rate of dead cells than the single and vehicle treated tumors (Figure 6D). In addition, proliferation was markedly reduced in tumors treated with the combination therapy as reflected by a reduced staining for Ki-67 (Figure 6D). Histological analysis showed no tissue alterations in brain, lung, kidney, heart, liver, spleen, intestine or pancreas that may indicate organo-toxic effects (Figure 6E).

## DISCUSSION

Evasion from apoptosis is a characteristic feature of cancer cells and is fundamental for drug resistance, especially in therapy refractory neoplastic diseases such as glioblastoma [14]. Therefore, it is of great importance to understand how apoptosis is being avoided and to develop strategies to overcome apoptotic resistance. Dysregulation of the Bcl-2 family proteins, resulting in an upward shift of the apoptotic threshold, is frequently encountered in cancer [8]. As a consequence therapeutic compounds (i.e. ABT737, ABT263) selectively targeting anti-apoptotic Bcl-2 family members were developed. However, Mcl-1 was found to confer resistance towards these agents [9, 10]. Therefore, a multi-targeting approach including Mcl-1-targeted BH3-mimetics seems like a valid strategy to overcome ABT-related drug resistance. Here, we describe a novel drug combination for the treatment of glioblastoma that consists of the orally available Bcl-2/Bcl-xL inhibitor ABT263 and the Mcl-1 inhibitor GX15-070. Both are currently being evaluated in clinical trials. In phase I clinical trials, single-agent treatment with ABT263 has been shown to have a favorable safety profile and to be well tolerated in patients with small-cell lung cancer or relapsed chronic lymphocytic leukemia [15-17]. Moreover, in a recently published phase II clinical trial, combined treatment with ABT263 and rituximab was reported to result in higher response rates when compared to treatment with rituximab alone in patients with previously untreated chronic lymphocytic leukemia [18]. Similarly, GX15-070 has been studied in phase I and phase II clinical trials for the treatment of patients with lymphoma, small-cell lung cancer, non-small-cell lung cancer and other solid tumors as a single-agent or in combination with different anti-neoplastic agents [19-22]. The treatment with GX15-070 was reported to be generally well tolerated and improved efficacy was stated by some of the studies [20, 21]. In our study, combined treatment with ABT263 and GX15-070 yielded a synergistic antiproliferative effect on gliobastoma cells. It is tempting to speculate whether our combination therapy may have a positive impact on standard of care treatments for glioblastoma, such as radiation and/or temozolomide. Since ABT263 and GX15-070 act at the level of the anti-apoptotic Bcl-2 family members it seemed promising to examine whether combined treatment enhances apoptosis. Consistently, established glioblastoma cell lines, stem cell-like glioma cells as well as primary cultures displayed hallmarks of apoptotic cell death, such as enhanced staining for annexin V, loss of mitochondrial membrane potential and activation of initiator and effector caspases. These findings are in accordance with the current literature [23-27].

While our initial hypothesis was that GX15-070 interferes with Mcl-1 activity, but not necessarily modulates its expression levels, we found that GX15-070 by itself markedly reduced the expression levels of Mcl-1 in glioblastoma cells, which in turn would be expected to attenuate ABT263-mediated up-regulation of Mcl-1 and thus drive susceptibility of glioma cells to BH3-mimetics. While Mcl-1 expression is known to be regulated through posttranscriptional and posttranslational mechanisms, there are also instances in which Mcl-1 is regulated at the level of transcription, e.g. through activating transcription factor 5 [28]. Therefore, we first assessed whether Mcl-1 mRNA levels are significantly affected by GX15-070 treatment. Our findings suggested that in glioblastoma cells there is a marked increase of Mcl-1 mRNA levels in response to increasing concentrations of GX15-070. However, this increase in mRNA did not correspond to the observed reduction of Mcl-1 protein. Thus, we hypothesized that GX15-070 affects Mcl-1 levels primarily posttranslationally. Several factors are known to interact with and stabilize Mcl-1 protein, e.g. Usp9X [13], Bag3 [12] and MULE [29]. GX15-070 did not greatly affect mRNA levels of Usp9X and even increased Bag3 mRNA expression however, protein levels were suppressed. This finding reinforced the notion that GX15-070 affects Mcl-1 protein levels through inhibition of its interacting partners, Usp9X and Bag3. While Usp9X belongs to the family of deubiquitinases and promotes Mcl-1 stability by removing ubiquitin chains from Mcl-1 [13], Bag3 most likely enhances Mcl-1 levels through its function as chaperone. However, both molecules drive Mcl-1 to high levels within tumor cells. Akin to Mcl-1, both Usp9X and Bag3 are expressed at higher levels in malignant tissues [30, 31]. Bag3 protein expression is increased in both low- and high-grade astrocytic gliomas, with the highest expression levels found in glioblastoma [30]. Knock-down of Bag3 caused apoptosis in a rat glioma model. Although Festa et al. have certainly established a role for Bag3 as a therapeutic target; they have not established Mcl-1 as a downstream mediator of Bag3 in malignant glioma. Moreover, GX15-070 has not been demonstrated before to modulate the expression levels of Bag3. Thus, to the best of our knowledge our present report is the first to highlight a regulation of Bag3 by GX15-070. In addition, drugs that modulate the expression or even specifically bind to Bag3 have not been described. However, given the potential therapeutic potency as demonstrated by our knock-down experiments of Bag3 and the fact that Bag3 is up-regulated in malignant tissue, it may be a worthwhile approach to unravel or design molecules targeting Bag3.

Concerning Usp9X, the present literature suggests that while Usp9X has a pivotal role during the development of the nervous system [32], its expression declines in the mature brain, suggesting that it is a specific therapeutic target for primary brain tumors. There is minimal information on Usp9X signaling in glioblastoma cells. Usp9X has not been targeted for clinical therapy in malignant glioma. Given that we and others demonstrated an apoptosis-inducing effect on glioblastoma cells subsequent to Usp9X-silencing [33], we believe that it is worthwhile considering Usp9X as a future therapeutic target. Thus far, it appears that Mcl-1 is one of the major effectors of Usp9X, but recent reports suggest that Usp9X may have further therapeutic implications. A recent study focused on Usp9X in the context of a frequent prostate carcinoma-related genetic translocation (TMPRSS2-ERG) [34] that facilitates tumor growth and thus not only represents a diagnostic aid, but also a potential therapeutic target. VCaP cells harbor the TMPRSS2-ERG gene fusion and in turn they express significant levels of ERG, which is stabilized by Usp9X. Interfering with Usp9X function by chemical inhibition through the deubiquitinase inhibitor, WP1130, results in proteasomal degradation of ERG and impaired growth of tumor cells exhibiting this genetic alteration *in vivo* [34]. Another compound that reduces Usp9X levels is Pemetrexed. While Usp9X was suppressed by Pemetrexed, the pro-apoptotic protein Noxa was inversely regulated. Based on this model system, Pemetrexed-mediated increase in Noxa resulted in suppression of Usp9X and subsequent depletion of Mcl-1 protein levels, rendering lung adenocarcinoma cells more sensitive to apoptotic stimuli. In our study, we demonstrate a novel mechanism by which GX15-070 elicits its anti-cancer properties beyond the inhibitory effects on Mcl-1. Our data show that GX15-070 was remarkably efficacious in suppressing Usp9X protein levels in glioblastoma cells. In the setting of the above mentioned studies it is tempting to speculate whether GX15-070 may be useful in other malignancies, in particular in advanced stage prostate cancer, displaying the TMPRSS2-ERG gene fusion. Another recent report links Usp9X to Beclin-1, a gene that is known as a tumor suppressor and a modulator of autophagy [35]. Elgendy et al. demonstrated that Beclin 1 interacts with Usp9X in a region related to the binding of Mcl-1 to Usp9X [35], linking Usp9X to both autophagy as well as intrinsic apoptosis. Although tumor cells harbored high levels of Mcl-1 and low levels of Beclin 1 – a relationship inversely found in normal cells – a more detailed understanding of how Usp9X regulates both apoptosis and autophagy is necessary.

Our results show that knock-down of both Bag3 and Usp9X leads to a depletion of Mcl-1 protein levels in glioblastoma cells and rendered them highly sensitive to ABT263. These findings are in line with reports from other groups, showing that knock-down of Usp9X or Bag3 sensitize tumor cells to ABT737. It is notable that our study is the first to demonstrate that single knock-down of both Bag3 and Usp9X are sufficient to sensitize glioblastoma cells to BH3-mimetics. Thus, targeting both Bag3 and Usp9X may represent novel approaches to overcome intrinsic and potentially extrinsic apoptotic resistance in glioblastoma. GX15-070 is unique in that it suppressed Bag3 and Usp9X levels simultaneously in glioblastoma cells independently of their *TP53* status, which most likely further enhanced GX15-070 mediated susceptibility to BH3-mimetics.

We further showed that the combination therapy of ABT263 and GX15-070 inhibited anchorage-independent growth of glioblastoma cells including therapy-resistant U87-EGFRvIII cells. Moreover, we found that the combination treatment significantly impaired *in vivo* growth of U87-EGFRvIII cells. These findings are promising as they suggest a proof-of-principle that the combined inhibition of Mcl-1-related chaperones in combination with selective Bcl-2/Bcl-xL inhibitors may be a suitable approach to combat the most resistant phenotypes of gliomas and potentially other entities.

Finally, we tested whether this drug combination is more effective than the single drugs alone in a heterotopic model of murine glioma. While animals receiving either ABT263 or GX15-070 revealed a subtle reduction in tumor growth, the combination treatment was significantly more active than the vehicle treated control, ABT263 or GX15-070 suggesting that this combined strategy exerts enhanced anti-glioma activity *in vivo*. These findings are consistent with previous studies, in which other compounds, such as mTOR/PI3K inhibitors [27] or Inhibitor of Apoptosis protein suppressors [36], were used to enhance the activity of BH3-mimetics *in vivo*. However, the approach presented here is unique in that our proposed combination therapy not only affects Mcl-1 itself, but also controls the expression levels of two major Mcl-1 interacting proteins, namely Usp9X and Bag3. We propose that compounds, affecting the Mcl-1 interacting protein family might be a welcome addition to the treatment repertoire for treatment resistant malignancies, such as glioblastoma.

## MATERIALS AND METHODS

### Ethics statement

All procedures were in accordance with Animal Welfare Regulations and approved by the Institutional Animal Care and Use Committee at the Columbia University Medical Center. The study was reviewed and approved by the institutional review board at the Columbia University Medical Center.

### Reagents

ABT263 and GX15-070 were from Selleckchem (Houston, TX, U.S.A.). A 10 mM working solution in dimethylsulfoxide (DMSO) was prepared for both reagents prior to storage at −20°C. Final concentrations of DMSO were below 0.1% (v/v).

### Cell cultures and growth conditions

U87MG, LN229, U373, U251 and T98G human glioblastoma cell lines were obtained from the American Type Culture Collection (Manassas, VA, U.S.A.). NCH644 and NCH421K stem cell-like glioma cells were obtained from Cell Line Services (CLS, Heidelberg, Germany). The identities of the glioblastoma cell lines we purchased were confirmed by the respective source of purchase except for U373, which is no longer verified by the ATCC. U87-EGFRvIII cells were kindly provided by Dr. Frank Furnari (Ludwig Institute for Cancer Research, La Jolla, CA, U.S.A.). GS9-6 [28] and 2927T2 are primary neurosphere stem-like glioma cells derived at the University of Massachusetts (Worcester, MA, U.S.A.). The 2927T2 line was derived from a recurrent tumor. The MGPP-3 (p53−/−, PTEN+/+) is a murine proneural glioblastoma cell which was kindly provided by Dr. Peter Canoll. All cells were cultured as previously described [37].

### Cell viability assays

In order to examine cellular proliferation, 3-[4, 5-dimethylthiazol-2-yl]-2, 5-diphenyltetrazolium bromide (MTT) assays were performed as previously described [37].

### Measurement of apoptosis and mitochondrial membrane potential

For annexin V APC staining the APC annexin V Apoptosis Detection Kit (BD Pharmingen, U.S.A.) was used according to the manufacturer's instructions. For PI staining, cells were resuspended in 300 μl PBS and fixated by adding 1000 μl ice-cold ethanol prior to incubation over night at 4ºC. Then the cells were centrifuged at 1800 rpm, the supernatant was removed and 400 μl PI/RNase staining solution (Cell signaling technology, Danvers, MA, U.S.A.) were added prior to incubation for 15 min at RT and flow cytometric analysis.

To detect intrinsic apoptosis staining for JC-1 was performed according to the manufacturer's instructions using the MitoProbe^TM^ JC-1 assay kit for flow cytometry (Molecular Probes^®^, Life Technologies) [38]. The data were analysed with the FlowJo software (version 8.7.1; Tree Star, Ashland, OR, U.S.A.).

### Soft agar assay

Anchorage-independent growth was examined as previously described [37]. Colonies with an area exceeding 625 μm^2^ were counted. Analysis was performed using the NIH ImageJ software (Bethesda, Maryland; http://imagej.nih.gov/ij).

### *In vivo* tumorigenicity assay

Effects on tumorigenesis *in vivo* were examined as previously described [39]. U87-EGFRvIII cells were treated with ABT263 (2 μM), GX15-070 (2 μM), the combination of both or solvent for 2 h prior to collection and subcutaneous implantation of 1 × 10^6^ cells into the flanks of 6-8 week-old SCID SHO mice. Tumor formation was closely monitored.

### Subcutaneous xenograft model

2 × 10^6^ U251 cells were implanted subcutaneously into the flanks of 6-8 week-old SCID SHO mice. Measurements were performed with a caliper and tumor sizes were calculated as (length x width^2^)/2. Treatment was performed intraperitoneally twice a week for 3 weeks. For intraperitoneal application ABT263 and GX15-070 were dissolved in 80% Cremophor EL (SIGMA, St. Louis, MO) and 20% Ethanol (Pharmco-Aaper, Brookfield, CT) (v/v).

### Western blot analysis

Specific protein expression in cell lines was determined by Western blot analysis as described before [27] using the following primary antibodies: Mcl-1 (1:500; CST: Cell Signaling Technology, Danvers, MA), human caspase-9 (1:1,000; CST), cleaved caspase-3 (1:250; CST), cleaved PARP (Asp214, 1:1000; CST), Bcl-xL (1:500; CST), Usp9X (1:1000; CST), Noxa (1:500, clone 114C307; Calbiochem), β-actin (1:2,000, clone AC15; Sigma Aldrich), Bag3 (1:500; Abcam, Cambridge, MA). 14-3-3 (1:1,000) and secondary HRP-linked antibodies were purchased from Santa Cruz Biotechnology.

### Transfections of siRNAs

SignalSilence^®^ Usp9X siRNA I #6308 was purchased from CST. Non-targeting siRNA-pool (ON-TARGETplus Non-targeting Pool, # D-001810-10-05) and siRNA against Bag-3 (SMARTpool: ON-TARGETplus Bag3 siRNA, L-011957-00-0005) and Mcl-1 (**SMARTpool: ON-TARGETplus Mcl-1 siRNA, L-004501-00-0005)** were purchased from Thermo Fisher Scientific (Pittsburgh, PA) and transfected as previously described [38].

### Real-time PCR and cDNA synthesis

RT-PCR was performed as described before [27] using the following primers: Usp9x forward: GTG TCA GTT CGT CTT GCT CAG C; Usp9X reverse: GCT GTA ACG ACC CAC ATC CTG A; Bag3 forward: TGC CAG AAA CCA CTC AGC CAG A; Bag3 reverse: TGA GGA TGA GCA GTC AGA GGC A; Mcl-1 forward: CCA AGA AAG CTG CAT CGA ACC AT; Mcl-1 reverse: CAG CAC ATT CCT GAT GCC ACC T; GAPDH forward: GTC TCC TCT GAC TTC AAC AGC G and GAPDH reverse: ACC ACC CTG TTG CTG TAG CCA A.

### Histological analysis

Subcutaneous tumors and samples from organs were extracted from SCID SHO mice and fixed for at least 24 hours in 10% PBS-buffered formalin. Then tissues were embedded in paraffin and 4 μm thick sections were cut prior to staining with hematoxylin and eosin. Microphotographs were taken at x40 magnification.

### Statistical analysis

Statistical significance was assessed by Mann-Whitney U-test using Prism version 5.04 (GraphPad, La Jolla, CA, U.S.A.). A p≤0.05 was considered statistically significant. Bliss analysis was performed to detect synergistic, additive or antagonistic effects as previously described [37].

## SUPPLEMENTARY MATERIAL FIGURES


