# Supplementary material for: Combined inhibition of Bcl-2/Bcl-xL and Usp9X/Bag3 overcomes apoptotic resistance in glioblastoma in vitro and in vivo
Source: Oncotarget. 2015 May 4;6(16):14507–21. doi: 10.18632/oncotarget.3993 (PMC4546483; doi:10.18632/oncotarget.3993)
Supplement: Supplementary file 1 [file oncotarget-06-14507-s001.pdf]

Combined inhibition of Bcl-2/Bcl-xL and Usp9X/Bag3 overcomes apoptotic resistance in glioblastoma *in vitro* and *in vivo*

Supplementary Material

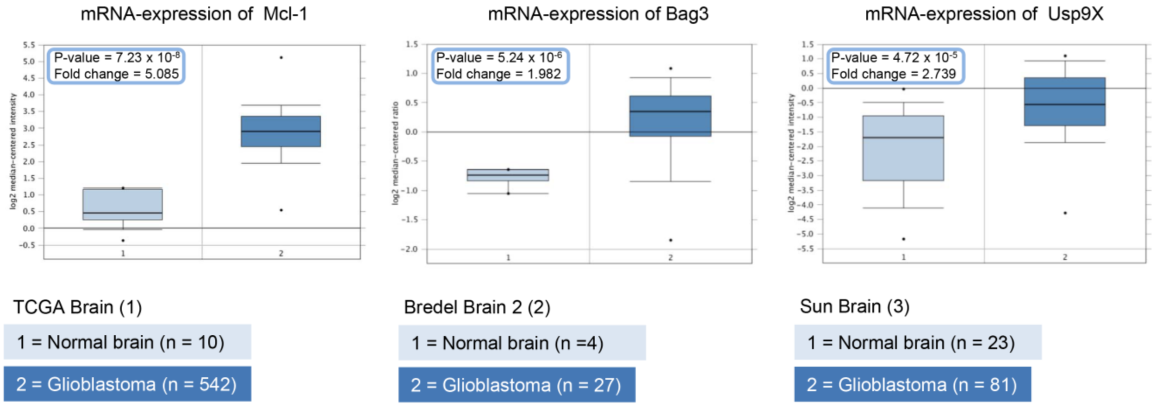

(1) The Cancer Genome Atlas - Glioblastoma Multiforme Gene Expression Data, http://tcga-data.nci.nih.gov/tcga/

(2) Bredel M et al. Cancer Res., 2005 65:8679-89

(3) Sun L et al. Cancer Cell, 2006 9:287-300

Suppl. fig. 1:

Mcl-1, Bag3 and Usp9X mRNA are overexpressed in glioblastoma compared to normal brain. *In silico* gene expression analysis of Mcl-1, Bag3 and Usp9X using the oncomine® database (www.oncomine.org, 01/2015, Compendia Bioscience, Ann Arbor, MI).

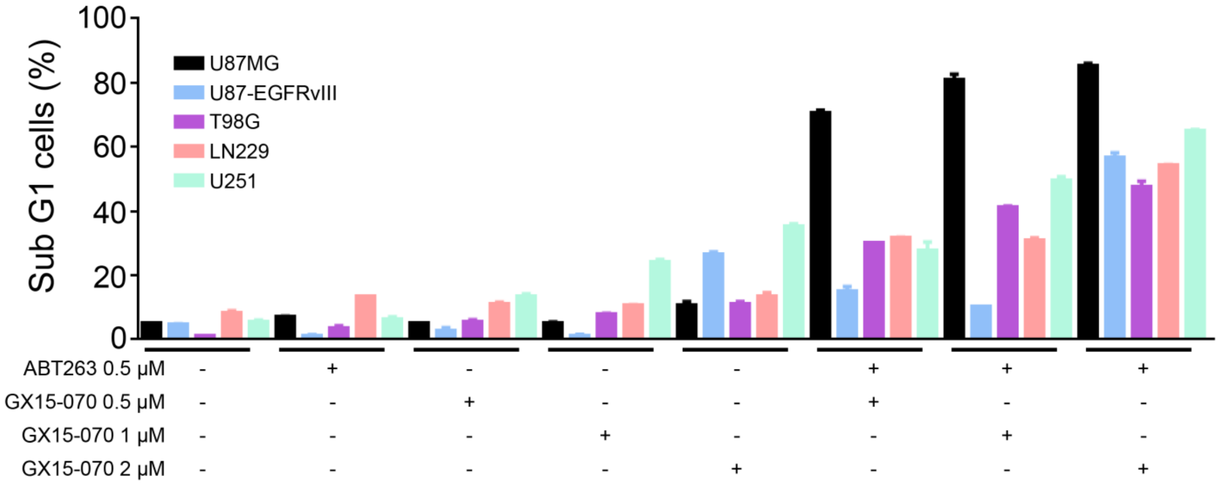

Suppl. fig. 2:

Combined treatment with ABT263 and GX15-070 yields enhanced induction of apoptosis. Quantitative representation of the fraction of sub G1 cells upon treatment of the indicated glioblastoma cell lines for 48 h with solvent, ABT263, increasing concentrations of GX15-070 or the respective combinations of ABT263 and GX15-070. *Columns*, means. *Bars*, SD.

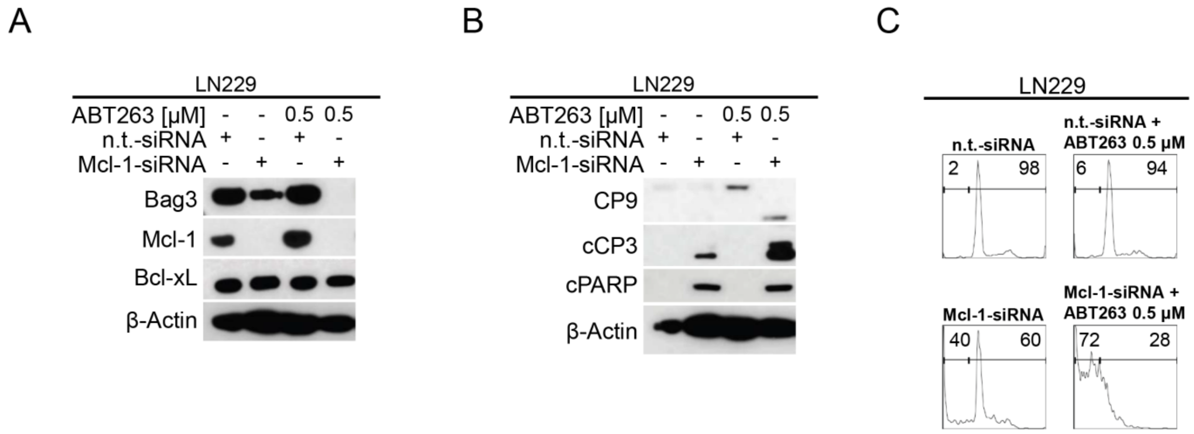

Suppl. fig. 3:

A, LN229 glioblastoma cells were treated with n.t.–siRNA or Mcl-1-siRNA prior to treatment with ABT263 at indicated concentrations for 7 h. Whole-cell extracts were examined by Western blot analysis for Bag3, Mcl-1, Bcl-xL. Actin served as a loading control. B, Western blots showing the expression of caspase 9 (CP9), cleaved caspase 3 (cC3) and cleaved PARP (cPARP) in LN229 glioblastoma cells treated as described for A. C, Representative histograms of LN229 glioblastoma cells treated with n.t.-siRNA or Mcl-1-siRNA prior to treatment with ABT263 for 24 h. Afterwards cells were stained for PI and subjected to flow cytometric analysis. The fraction of sub-G1 cells was determined.
